# The effect of excess fluid balance on the mortality rate of surgical patients: a multicenter prospective study

**DOI:** 10.1186/cc13151

**Published:** 2013-12-10

**Authors:** João M Silva, Amanda Maria Ribas Rosa de Oliveira, Fernando Augusto Mendes Nogueira, Pedro Monferrari Monteiro Vianna, Marcos Cruz Pereira Filho, Leandro Ferreira Dias, Vivian Paz Leão Maia, Cesar de Souza Neucamp, Cristina Prata Amendola, Maria José Carvalho Carmona, Luiz M Sá Malbouisson

**Affiliations:** 1 Hospital do Servidor Público Estadual-SP, R. Pedro de Toledo, 1800, Vila Clementino, São Paulo, SP 04039-004, Brazil; 2 Hospital das Clinicas SP-FMUSP, Anesthesiology Department, Av. Dr. Enéas de Carvalho Aguiar, 255, Cerqueira César, São Paulo, SP 05403-000, Brazil; 3 Hospital do Câncer de Barretos, Rua Antenor Duarte Vilela, 1331 - Doutor Paulo Prata, Barretos, SP 14780-000, Brazil; 4 Faculdade de Medicina da Universidade de São Paulo (FMUSP), Anesthesiology Postgraduate, Av. Dr. Arnaldo, 455, Cerqueira Cesar, São Paulo, SP 01246-903, Brazil

## Abstract

**Introduction:**

In some studies including small populations of patients undergoing specific surgery, an intraoperative liberal infusion of fluids was associated with increasing morbidity when compared to restrictive strategies. Therefore, to evaluate the role of excessive fluid infusion in a general population with high-risk surgery is very important. The aim of this study was to evaluate the impact of intraoperative fluid balance on the postoperative organ dysfunction, infection and mortality rate.

**Methods:**

We conducted a prospective cohort study during one year in four ICUs from three tertiary hospitals, which included patients aged 18 years or more who required postoperative ICU after undergoing major surgery. Patients who underwent palliative surgery and whose fluid balance could change in outcome were excluded. The calculation of fluid balance was based on preoperative fasting, insensible losses from surgeries and urine output minus fluid replacement intraoperatively.

**Results:**

The study included 479 patients. Mean age was 61.2 ± 17.0 years and 8.8% of patients died at the hospital during the study. The median duration of surgery was 4.0 (3.2 to 5.5) h and the value of the Simplified Acute Physiology Score (SAPS) 3 score was 41.8 ± 14.5. Comparing survivors and non-survivors, the intraoperative fluid balance from non-survivors was higher (1,950 (1,400 to 3,400) mL vs. 1,400 (1,000 to 1,600) mL, *P* <0.001). Patients with fluid balance above 2,000 mL intraoperatively had a longer ICU stay (4.0 (3.0 to 8.0) vs. 3.0 (2.0 to 6.0), *P* <0.001) and higher incidence of infectious (41.9% vs. 25.9%, *P* = 0.001), neurological (46.2% vs. 13.2%, *P* <0.001), cardiovascular (63.2% vs. 39.6%, *P* <0.001) and respiratory complications (34.3% vs. 11.6%, *P* <0.001). In multivariate analysis, the fluid balance was an independent factor for death (OR per 100 mL = 1.024; *P* = 0.006; 95% CI 1.007 to 1.041).

**Conclusions:**

Patients with excessive intraoperative fluid balance have more ICU complications and higher hospital mortality.

## Introduction

Volume management in the perioperative period has played a pivotal role in morbidity and mortality in surgical patients even though it receives less attention nowadays [1,2].

Fluid administration is generally accepted as doctrine for resuscitation of critically ill patients in many clinical circumstances including major surgery, shock and trauma. The biological rationale for such therapy is that fluid losses should be replaced to maintain homeostasis in order to prevent organ hypoperfusion and subsequent organ dysfunction [3,4].

Volume management in surgical patients is dictated by the volume status, maintenance fluid requirements, insensible losses and losses to the extravascular space [5-7]. However, hemodynamic changes (vasodilatation and myocardial depression), and primary or secondary inflammatory changes (increased vascular permeability) are also factors that can influence the volume administered [8].

On the other hand, emerging data show that the type, timing and amount of fluid may affect the clinical outcome. Thus, synthetic colloids can increase the risk of acute kidney injury [9], the administration of early fluid therapy in sepsis may improve survival [10], and delayed fluid therapy in patients with acute lung injury may increase the duration of mechanical ventilation [11]. In addition, the accumulated positive balance probably contributes to increased morbidity and mortality [12-16].

Therefore, the aim of this study was to evaluate the impact of intraoperative fluid balance on the postoperative organ dysfunction, infection and mortality rate.

## Method

After approval by the Ethics and Research Committees from Clínicas Hospital (São Paulo - SP), Servidor Publico Hospital (São Paulo - SP) and the Cancer Hospital (Barretos-SP) and registering the study in the National System of Information about Ethics in Research, a written post-informed consent was obtained from each patient or legal representative, and the study was conducted in three tertiary hospitals. It was an observational study whose inclusion criteria were patients aged ≥18 years undergoing surgery that required postoperative ICU.

Exclusion criteria included patients undergoing palliative surgery, with short life expectancy, patients with renal failure, patients with NYHA class IV heart failure or ejection fraction on echocardiography less than 30%, patients with diagnosis of diabetes mellitus (prior diabetic diagnosis or after fasting for at least eight hours and two perioperative glucose readings higher than 126 mg/dl) [17] and those who refused to participate in the study. Exclusions of the clinical conditions above were established because of their influence on fluid balance, potentially interfering with the study analyses.

The preoperative maintenance of fluid used 10% glucose solution to supply 2 g/kg/day of caloric intake [18] due to preoperative fasting, but it was not added to the calculation of the fluid balance, as well as blood loss intraoperatively, because it would be very difficult to standardize the calculation about blood loss among the centers. In addition, the colon preparation was only accomplished in 0.63% (n = 3) of the patients involved in the study; for this reason they also were not quantified in the fluid balance.

The calculation of the fluid balance was considered as the sum of crystalloids, colloids used during surgery minus the sum of urine output plus 2 ml/kg/h during preoperative fasting plus intraoperative insensible losses (third space losses) of 1 to 2 mL/Kg/h in patients undergoing orthopedic, vascular and neurological surgery, 2 to 4 mL/kg/h in thoracic surgery and abdominal surgery without bowel exposure and 5 mL/kg/h in those undergoing surgery with bowel exposure. However, the maximum of 5 mL/kg/h was considered between preoperative fasting and intraoperative insensible losses even if the sum had been higher than 5 mL/kg/h [5-7,18,19]. According to the anesthetist evaluation, the criterion to guide intraoperative fluid replacement considered hemodynamic parameters such as blood pressure, urine output and heart rate. Postoperatively, the clinician had, as a goal, the improvement of perfusion parameters.

All patients were followed during the remainder of their hospital stay and 90 days after they were discharged. Postoperative organ dysfunction was evaluated as: cardiovascular (need for vasoactive drugs or vasodilator for more than one hour despite fluid resuscitation), respiratory (partial pressure of oxygen in the blood/fraction of inspired oxygen (PaO_2_/FiO_2_) <200, reintubation, difficulty in withdrawal of mechanical ventilation in the postoperative period), renal failure (creatinine increase by 50% or urine output less than 400 ml in 24 hours), neurological (behavior change, forgetfulness or psychomotor agitation) and coagulation (platelet decrease by 30% from baseline). Furthermore, ICU infections, length of ICU and hospital stay were also evaluated. The criteria used for the diagnosis of infection were made and revised according to Centers for Disease Control (CDC) guidelines [20].

SAPS 3 was evaluated at the time of inclusion [21], and the worst values of the variables at this time were used along with the American Anesthesiology Society (ASA) physical status classification system [22].

Finally, the patients were divided into two groups: survivors and non-survivors. The analysis of sensitivity and specificity considered the value of fluid balance with best accuracy for hospital mortality, the value was chosen by *Youden's index* (sensitivity + specificity -1). This value was used as the cut-off point to consider excessive fluid balance or not, and to evaluate complications and outcomes after surgery.

### Statistical analysis

Initially the demographic, clinical and physiological features of patients included in this study were described. For the description of categorical variables the frequencies and percentages were calculated. Quantitative variables were described using central tendency and dispersion measures.

The choice of the statistical method used in assessing each variable was based on their distribution pattern. The categorical variables were analyzed by the chi-square test and the continuous variables by the mean with the Student's *t*-test. Continuous variables with irregular distribution were analyzed by the Mann-Whitney Test. Values of *P* <0.05 were considered significant. The Statistical Package for Social Sciences (SPSS) (Faculdade de Medicina da Universidade de São Paulo (FMUSP), São Paulo, Brazil) 20.0 was used for analysis of such calculations. Initially, the patients from the survivors’ group were compared to patients from the non-survivors’ group and, subsequently, the secondary outcomes were compared from a value of fluid balance according to the analysis of sensitivity and specificity for hospital mortality.

A binary logistic regression analysis was also performed applying stepwise selection with backward elimination in order to identify independent risk factors and control confounding effects (variables mutually adjusted). Variables with significant probability (*P*-value) less than 0.05 were considered as candidates, and they were removed in each step if they presented probability (*P*-value) higher or equal to 0.10. Afterward, the selected variables for the regression model were tested to evaluate pairwise interaction possibilities, and those variables with interactions were corrected in the main regression model. A bootstrap procedure based on 10,000 bootstrap samples was applied in the regression model to investigate the stability of coefficients and predictive ability of the variables included in model. In addition, to verify any possible confounding effects of variability in the clinical practice from the three centers, a dummy variable about centers was included in the main regression model. The estimated cumulative 90-day survival was observed using a Kaplan-Meier curve.

## Results

The study included 479 patients (mean age 61.2 years, 51.1% male). The majority of the patients were ASA II and the most prevalent surgery was of the gastrointestinal tract. While still in the hospital, 8.8% of the patients died. Comparing survivors and non-survivors, there was a statistically significant difference upon univariate analysis for male subjects, those with high SAPS 3, ASA II and III, patients with higher fluid balance during surgery and those requiring vasopressors and blood transfusions intraoperatively (Table 1).

**Table 1:** Comparison of survivors and non-survivors

**Variables**	**All patients (n = 479)**	**Survivors (n = 437)**	**Non-survivors (n = 42)**	***P***
Age (years)	64.0 (51.0 to 74.0)	63.0 (51.0 to 74.0)	67.0 (46.0 to 77.0)	0.459
Male gender (%)	51.1	49.4	62.9	0.047
SAPS 3	41.8 ± 14.5	40.6 ± 14.2	53.8 ± 12.3	<0.001
ASA (%)				
I	13.4	13.5	11.9	0.96
II	52.8	55.8	21.4	<0.001
III	27.8	26.3	42.9	0.047
Pre-operative time fasting (h)	15.5 (10.2 to 18)	16 (10.2 to 18)	14.6 (10 to 17.5)	0.43
**Kind of surgery**				
Gastrointestinal surgery (%)	34.3	34.4	32.5	0.94
Vascular surgery (%)	14.5	13.5	25.0	0.07
Orthopaedic surgery (%)	12.8	13.8	2.5	0.06
Others* (%)	38.4	38.3	40.0	0.96
**Anesthesia (%)**				0.432
General	55.5	55.5	56.0	
Neuroaxis	31.8	32.5	24.0	
General + Neuroaxis	12.7	12.0	20.0	
Surgery time (hours)	4.0 (3.2 to 5.5)	4.0 (3.0 to 5.5)	4.0 (3.3 to 6.0)	0.76
Elective surgery (%)	95.5	94.9	94.1	0.887
Crystalloid intraoperatively (mL)	3,500.0 (2,000.0 to 6,500.0)	3,500.0 (2,000.0 to 6,500.0)	4,500.0 (2,375.0 to 8,250.0)	0.11
Colloid intraoperatively (mL)	500.0 (500.0 to 1,000.0)	500.0 (250.0 to 500.0)	500.0 (500.0 to 1,000.0)	0.10
Fluid balance intraoperatively (ml)	1,400.0 (1,000.0 to 2,000.0)	1,400.0 (1,000.0 to 1,600.0)	1,950.0 (1,400.0 to 3,400.0)	<0.001
Transfusion requirements intraoperatively (%)	24.8	23.1	43.2	0.007
Need for vasopressors intraoperatively (%)	56.6	54.5	78.4	0.005
Lactate at end of surgery (mmol/L)	2.4 ± 1.6	2.4 ± 1.6	2.6 ± 1.5	0.459

*Others: neurosurgery, head and neck surgery, thoracic surgery, urologic surgery, gynecologic surgery. All values between parentheses represent percentile 25 to 75 values observed; all values represent mean ± standard deviation.

However, the independent variables that influenced hospital mortality were SAPS 3, ASA and high intraoperative fluid balance. The regression model found good area under the Receiver Operating Characteristic (ROC) (AUC) equal 0.835 and 95% confidence interval (CI) 0.799 to 0.868 (Table 2).

**Table 2:** Independent variables for hospital mortality

	***P***	**OR**	**95% CI**		**Bootstrap**	
					**95% CI**	
			**Lower**	**Upper**	**Lower**	**Upper**
SAPS 3 (per unit)	<0.001	1.050	1.026	1.074	1.055	1.145
ASA (per unit)	0.002	1.892	1.276	2.806	1.400	4.113
Fluid balance intraoperatively (per 100 ml)	0.006	1.024	1.007	1.041	1.014	1.093

The variables were adjusted in model by gender, SAPS 3, ASA, transfusion, fluid balance and vasopressors intraoperatively. Unless otherwise stated, the bootstrap results are based on 10,000 bootstrap samples.

In addition, in logistic regression even adding the centers to verify a possible confounding effect of variability in clinical practice from the three hospitals, it found that SAPS 3 (OR = 1.050; 95% CI 1.026 to 1.074), ASA (OR = 1.919; 95% CI 1.295 to 2.844) and high intraoperative fluid balance per 100 mL (OR = 1.025; 95% CI 1.009 to 1.042) still remained independent variables from death.

**Figure 1:**
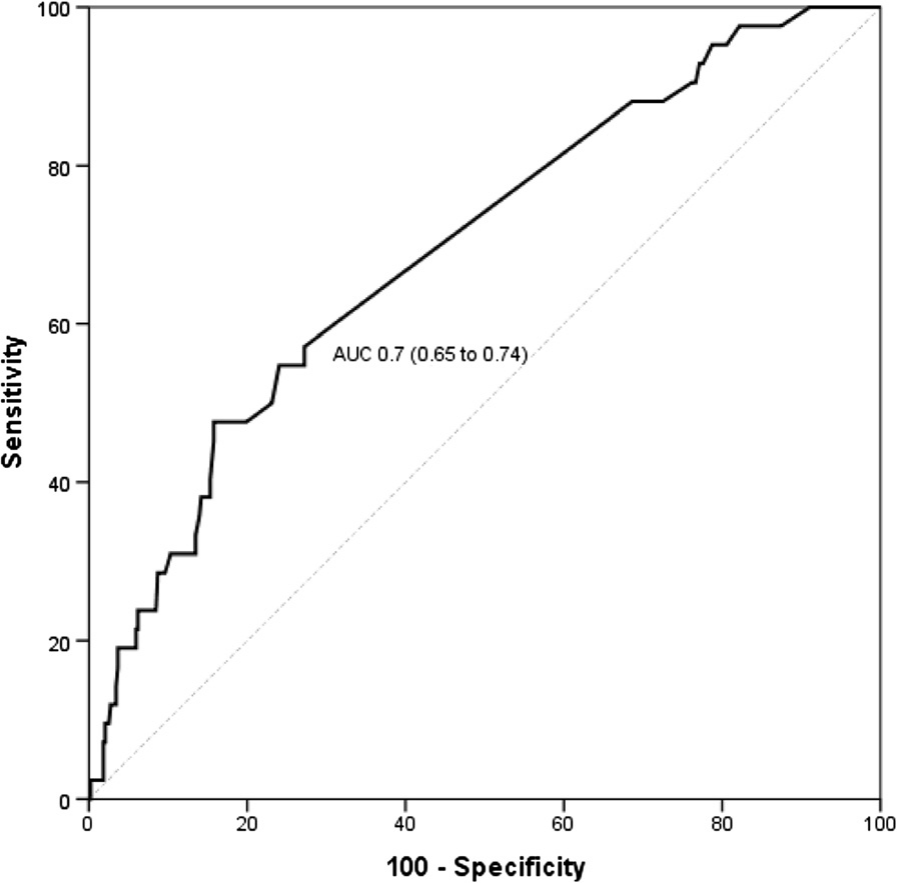
Fluid balance ROC curve for prediction of hospital mortality.

To determine the best cut-off point to discriminate excessive from non-excessive fluid balance, tests of sensitivity and specificity were performed correlating hospital death and fluid balance. The area under the ROC was 0.7 (0.65 to 0.74) and the optimal fluid balance value found to discriminate hospital mortality was 2,000 mL (sensitivity of 47.62% and specificity of 84.21%; Figure 1). Patients with excessive fluid balance had higher hospital mortality compared to the other patients (18.7% vs. 5.9%, *P* <0.001).

The analyses of the outcomes after surgery revealed that patients with excess fluid balance intraoperatively had a longer ICU stay (4.0 (3.0 to 8.0) vs. 3.0 (2.0 to 6.0), *P* <0.001) and higher incidence of infectious (41.9% vs. 25.9%, *P* = 0.001) and had higher postoperative organ dysfunctions: neurological (46.2% vs. 13.2%, *P* <0.001), cardiovascular (63.2% vs. 39.6%, *P* <0.001) and respiratory (34.3% vs. 11.6%, *P* <0.001). Furthermore, interestingly, urine output in the first 24 hours postoperatively was lower in these patients (Table 3).

**Table 3:** Comparison of patients with or without excessive fluid balance

**Variables**	**All patients (n = 479)**	**Fluid balance is not excessive (n = 372)**	**Fluid balance is excessive (n = 107)**	***P***
Postoperative organ dysfunction (%)	61.6	57.1	77.4	<0.001
Cardiovascular	44.9	39.6	63.2	<0.001
Neurological	20.5	13.2	46.2	<0.001
Respiratory	16.6	11.6	34.3	<0.001
Renal	20.0	19.9	20.0	0.990
Coagulation	12.6	12.4	13.2	0.825
Urine output in the first postoperative 24 hours (mL)	1,250.0 (800.0 to 2,000.0)	1,300.0 (800.0 to 2,100.0)	1,050.0 (700.0 to 1,750.0)	0.034
Infection	29.4	25.9	41.9	0.001
Days of mechanical ventilation	3.0 (1.0 to 7.0)	3.0 (1.0 to 6.0)	3.0 (1.0 to 7.0)	0.659
ICU stay (days)	4.0 (2.0 to 7.0)	3.0 (2.0 to 6.0)	4.0 (3.0 to 8.0)	<0.001
Hospital stay (days)	15.0 (8.0 to 26.0)	15.0 (8.0 to 25.7)	15.0 (8.0 to 26.0)	0.809

All values between parentheses represent percentile 25 to 75 values observed; all values represent mean ± standard deviation.

**Figure 2:**
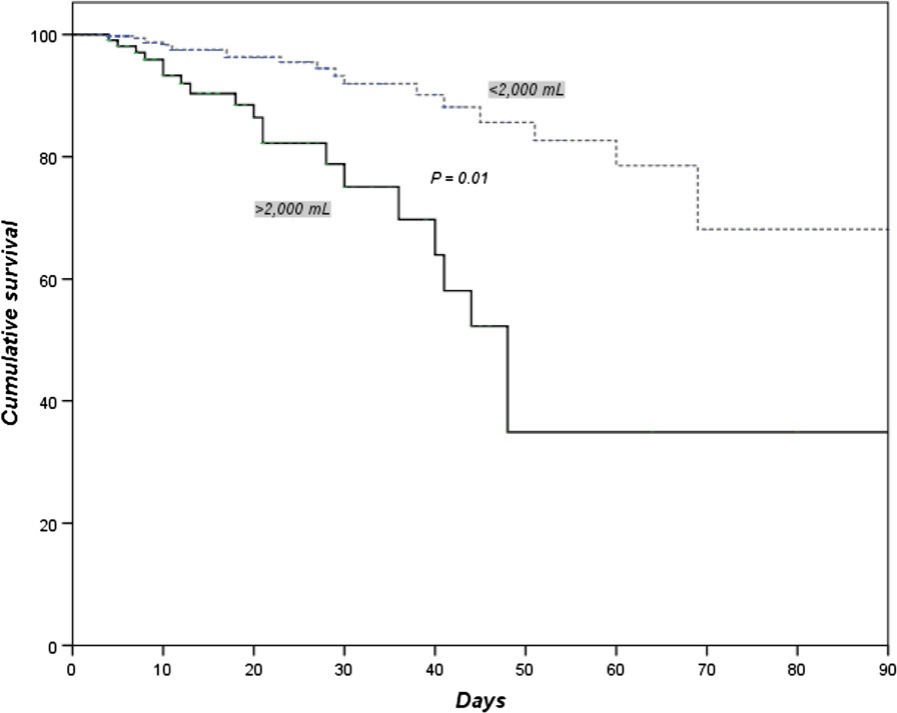
Kaplan-Meier curve among patients with or without excessive fluid balance up to 90 days.

In the assessment by the Kaplan-Meier method, there was a statistically significant difference in patient survival up to 90 days. Patients with excessive fluid balance showed a lower survival rate (Figure 2).

## Discussion

The present study demonstrates higher organ (especially cardiovascular, neurological and respiratory) dysfunction and infection in the ICU in patients with excessive intraoperative fluid balance. It is noteworthy that this study was multicenter and involved a general population of high-risk surgery. Therefore, fluid balance can be overestimated for some surgeries.

The current clinical practice for fluid administered in the perioperative period remains controversial. A comparison of 19 different studies showed that fluid replacement included in early hemodynamic optimization improves the prognosis of surgical patients [23].

However, fluid overload and saline consequences have been shown in the literature [24], which eliminates the preference for a liberal fluid management. The adverse effects of volume overload are more evident in the lungs, where fluid resuscitation can lead to acute pulmonary edema compromising gas exchange and making the patients more susceptible to infections. Gastrointestinal tract edema increases postoperative ileus and gastric emptying times, and reduces lymphatic drainage and oxygenation, consequently impairing anastomotic healing [25]. Thus, the evaluation of intraoperative fluid balance can contribute to a restrictive strategy of fluid perioperatively.

Overall, volume overload results in tissue and interstitial edema, leading to poor diffusion of oxygen and metabolites, distortion of tissue architecture with obstruction of capillary blood flow and lymphatic drainage, and disorders of the interaction between cells. All these factors contribute to progressive organ dysfunction. The effects are even more pronounced in encapsulated organs such as the kidneys and liver because of their limited ability to accommodate additional volumes without increasing interstitial pressure and impairing blood flow [26,27].

Myocardial edema may worsen ventricular function, resulting in deterioration of oxygen supply and cardiac conduction [28]. Excessive intraoperative volume can also lead to increased demand for cardiac function, displacing the heart’s Starling curve and culminating in increased cardiac morbidity. Indeed, in the current study, it was determined that patients with excessive fluid balance presented more cardiovascular problems.

Our findings suggest, in agreement with Shields *et al*. [29], that there is a close connection between excessive intravascular volume and increased mortality, morbidity and length of hospital stay in this population.

Other studies have compared liberal versus restrictive fluid administration to clarify the best perioperative management. However, the development of guidelines becomes a difficult task since scientific evidence with multicenter randomized trials is rare and not consensual about the optimal amount of fluid in patients [29]. Hence, there is a varied regimen of fluid replacement without a uniform definition of what is restrictive and liberal, as well as variations in the timing of the perioperative period studied. For this reason, our study has relevance.

Nisanevich *et al*. [2] compared groups of patients submitted to abdominal surgery who received 4 or 12 ml/kg of hydration. The strict regime was accompanied by drastic reduction of hospitalization and time to lung recovery. The patients also had fewer complications and moderate weight gain postoperatively.

Lobo *et al*. [30] evaluated the postoperative period and reported a decrease in the incidence of complications, especially gastrointestinal, and shorter hospital stay in patients who were restricted to ≤2 L per day of crystalloid solution when compared to patients who received the standard regimen of 3 L per day.

Evidence suggests that a positive fluid balance of 5 to 10% of body weight gain is associated, in critically ill patients, with worse organ dysfunction and prognosis in the postoperative period of elective surgeries. Furthermore, in this study it was verified that urine output in the first 24 hours postoperatively in patients with excessive fluid balance was worse than in other patients. There is no evidence suggesting that the positive fluid balance brings any benefit for renal function [27].

Another study by Holte *et al*. [31] in patients who underwent laparoscopic cholecystectomy demonstrated the superiority of a restrictive over a liberal regime only in the preservation of lung function and hypoxemia, without any other benefit.

This study has limitations, including the fact that we did not assess the postoperative fluid management, as well as fluid expansion and fluid maintenance intraoperatively were not separately evaluated, because our proposal was that the study represented a real clinical practice, which does not invalidate our results considering the statistical analysis applied. Moreover, this was not the goal of the study, which was based on intraoperative fluid balance. Besides, the fluid balance mensuration was estimated instead of directly measured by, for example, weight gain. Furthermore, we only considered fluid balance rather than the amount of fluid infusion. This can be explained by the fact that each type of surgery has different characteristic intraoperative fluid losses and they could require different volumes. In spite of the study limitations, there are only a few multicenter studies that have evaluated or compared intraoperative infusion resuscitation strategies in a general population of high-risk surgeries. Although some current trends are for a restrictive practice [25,32], further studies are still needed to consolidate this issue.

Other limitations should be considered, such as the exclusion of diabetic patients since they are often subjected to major surgery; however, these patients could present an imbalance that directly affects the calculation of the fluid balance; it means they could have a higher uncompensated metabolic probability at any point during surgery, which could affect fluid replacement and hence the fluid balance calculation [17]. This study also did not consider blood loss in fluid balance; this option was based on variations that may occur among observers in computing blood loss. Another problem was the absence of a protocol to guide the intraoperative volume expansion, because it could be different for real clinical practice, but it was minimized by common agreement to follow hemodynamic parameters including blood pressure, heart rate and urine output, according to the anesthetists’ team decision.

## Conclusions

Patients with excessive intraoperative fluid balance have more postoperative organ dysfunction, more infections, and higher length of ICU stay and hospital mortality.

## Key messages

● This multicenter observational study with 479 surgical high-risk patients showed that excessive fluid balance may determine a higher postoperative mortality rate.

● Excessive fluid balance was independently associated with a higher risk of death.

● Patients who received excessive fluid balance intraoperatively had higher incidence of postoperative organ (mainly cardiovascular, respiratory and neurologic) dysfunction and ICU infection.

● The length of ICU stay was higher in patients with excessive fluid balance intraoperatively.

## Abbreviations

ASA: American Anesthesiology Society; CDC: Centers for disease control; CI 95%: 95% confidence interval; ICU: Intensive care unit; NYHA: New York Heart Association Functional Classification; OR: Odds ratio; ROC: Receiver operating characteristic; SAPS: 3 score simplified acute physiology score 3; SPSS: Statistical package for social sciences.

## Competing interests

The authors declare that they have no competing interests. No financial support was received for this study.

## Authors' contributions

JMSJR conceived of this study, participated in the design of the study, performed the statistical analysis and drafted the manuscript. FAMN, PMMV, MCPF, CSN, VPLM and CPA performed the collection of data. LMSM performed the statistical analysis, helped in the interpretation of data and drafted the manuscript. FAMN, AMRRO, LFD and LMSM helped in revising the draft of the manuscript. MJCC and LMSM helped in the final revision and writing of the manuscript. All authors read and approved the final manuscript.
